# The Bone-Cardiovascular Axis: Mechanisms and Clinical Relevance

**DOI:** 10.1155/2018/9689106

**Published:** 2018-02-21

**Authors:** Nicolas Verheyen, Martin R. Grübler, Cristiana Catena, Astrid Fahrleitner-Pammer, Adriana J. van Ballegooijen

**Affiliations:** ^1^Department of Cardiology, Medical University of Graz, Graz, Austria; ^2^Division of Endocrinology and Diabetology, Department of Internal Medicine, Medical University of Graz, Graz, Austria; ^3^Department of Cardiology, Swiss Cardiovascular Center Bern, Bern University Hospital, University of Bern, Bern, Switzerland; ^4^Hypertension Unit, Internal Medicine, Department of Experimental and Clinical Medical Sciences, University of Udine, Udine, Italy; ^5^Department of Internal Medicine, Division of Endocrinology and Diabetology, Medical University of Graz, Graz, Austria; ^6^Department of Health Sciences, Vrije Universiteit Amsterdam and the Amsterdam Public Health Research Institute, Amsterdam, Netherlands

Both bone and cardiovascular disease (CV) are leading causes of morbidity and mortality worldwide and particularly in ageing Western societies. Their common coincidence has been largely attributed to shared risk factors; however, increasing evidence also points towards direct mechanistic interweavement between bone metabolism, the vasculature, and the heart [1–4]. Direct and indirect crosslinks appear to become pathophysiologically relevant in the presence of specific comorbidities associated with imbalances in mineral and bone metabolism or the renin-angiotensin-aldosterone system (RAS) [5–7]. Under the umbrella of this special issue, experts in the field provide a broad and up-to-date overview and novel insights into hormonal interactions underlying the bone-cardiovascular axis.

A. Zittermann comprehensively reviews the role of vitamin D in bone and CV disease particularly stressing the great need for studies investigating the effects of vitamin D on CV health in patients with vitamin D deficiency.

Enriching the vitamin D discussion, A. J. van Ballegooijen et al. review the novel and increasingly important chapter on the mutual relationship between vitamins D and K and the impact of this interaction for both bone and CV health stating that optimal concentrations of both vitamins are needed to function properly.

In addition to these classical mineral hormones, the impact of hormones of the RAS, such as angiotensin II or aldosterone, on bone and CV health has recently attracted attention. C. Catena et al. provide an overview on the existing literature. They conclude that high aldosterone appears to be harmful particularly in concomitance with high salt intake and high parathyroid hormone (PTH).

In fact, there is a broad basis in the literature suggesting that interaction between the CV risk modifier PTH and the RAS is crucial for the development of bone and CV disease [8, 9]. S. Zaheer et al. extend this existing knowledge by showing that ACE inhibition leads to a reduction of PTH levels in patients with but not in patients without primary hyperparathyroidism.

Chronic kidney disease (CKD) is another specific condition where bone and cardiovascular disease are closely interweaved as a consequence of CKD-related mineral and bone disorders (CKD-MBD). In a novel murine model introduced by B. Frauscher et al., brown non-Agouti mice fed with high-phosphate diet developed media calcification, secondary hyperparathyroidism, and low-turnover bone disease. This novel noninvasive model will provide the opportunity to investigate the bone-cardiovascular axis related with CKD-MBD.

Finally, the important aspect of gender differences in the clinical relevance of the bone-cardiovascular axis finds further substrate in epidemiological analyses of two German cohort studies (*n* = 5680) reported by V. Lange et al. The authors found significant associations between presence of carotid plaques and quantitative ultrasound parameters of the heel only in males and stress the importance of screening for cardiovascular disease in males with osteoporosis.

Conclusively, basic and clinical evidence on the bone-cardiovascular axis is growing, while the clinical relevance is only at the beginning to become unveiled and much remains to be done. This issue shall provide insights into the exciting and complex mechanisms underlying the bone-cardiovascular axis and open the reader's mind towards novel and innovative hypotheses that will contribute to the future shape of this research field. It is also intended to motivate researchers to further investigate the clinical relevance of the bone-cardiovascular axis in order to improve guidance for the prevention and treatment of the still unacceptably high burden of bone and CV disease.

We, the Editorial Team, have been delighted to lead this special issue and hope that the readership will enjoy reading it.

*Nicolas Verheyen* *Martin R. Grübler* *Cristiana Catena* *Astrid Fahrleitner-Pammer* *Adriana J. van Ballegooijen*
